# Patient Ability and Willingness to Participate in a Web-Based Intervention to Improve Hypertension Control

**DOI:** 10.2196/jmir.1625

**Published:** 2011-01-20

**Authors:** Beverly B Green, Melissa L Anderson, James D Ralston, Sheryl Catz, Paul A Fishman, Andrea J Cook

**Affiliations:** ^5^Department of BiostatisticsUniversity of WashingtonSeattle, WAUnited States; ^4^School of Public HealthUniversity of WashingtonSeattle, WAUnited States; ^3^Group Health PermanenteSeattle, WAUnited States; ^2^Medical SchoolUniversity of WashingtonSeattle, WAUnited States; ^1^Group Health Research InstituteSeattle, WAUnited States

**Keywords:** electronic medical record

## Abstract

**Background:**

Patient-shared electronic health records provide opportunities for care outside of office visits. However, those who might benefit may be unable to or choose not to use these resources, while others might not need them.

**Objective:**

Electronic Communications and Home Blood Pressure Monitoring (e-BP) was a randomized trial that demonstrated that Web-based pharmacist care led to improved blood pressure (BP) control. During recruitment we attempted to contact all patients with hypertension from 10 clinics to determine whether they were eligible and willing to participate. We wanted to know whether particular subgroups, particularly those from vulnerable populations, were less willing to participate or unable to because they lacked computer access.

**Methods:**

From 2005 to 2006, we sent invitation letters to and attempted to recruit 9298 patients with hypertension. Eligibility to participate in the trial included access to a computer and the Internet, an email address, and uncontrolled BP (BP ≥ 140/90 mmHg). Generalized linear models within a modified Poisson regression framework were used to estimate the relative risk (RR) of ineligibility due to lack of computer access and of having uncontrolled BP.

**Results:**

We were able to contact 95.1% (8840/9298) of patients. Those refusing participation (3032/8840, 34.3%) were significantly more likely (*P* < .05) to be female, be nonwhite, have lower levels of education, and have Medicaid insurance. Among patients who answered survey questions, 22.8% (1673/7354) did not have computer access. Older age, minority race, and lower levels of education were risk factors for lack of computer access, with education as the strongest predictor (RR 2.63, 95% CI 2.30-3.01 for those with a high school degree compared to a college education). Among hypertensive patients with computer access who were willing to participate, African American race (RR 1.22, 95% CI 1.06-1.40), male sex (RR 1.28, 95% CI 1.18-1.38), and obesity (RR 1.53, 95% CI 1.31-1.79) were risk factors for uncontrolled BP.

**Conclusion:**

Older age, lower socioeconomic status, and lower levels of education were associated with decreased access to and willingness to participate in a Web-based intervention to improve hypertension control. Failure to ameliorate this may worsen health care disparities.

**Trial Registration:**

Clinicaltrials.gov NCT00158639; http://www.clinicaltrials.gov/ct2/show/NCT00158639 (Archived by WebCite at http://www.webcitation.org/5v1jnHaeo)

## Introduction

There is increasing evidence that patient access to practice-based electronic health records (defined here as patient-shared electronic health records) [1], combined with secure Web-based communications between patients and health care providers, improves the treatment of chronic diseases [2,3], and may result in improved health outcomes and decreased costs [4,5]. Their use is consistent with the Institute of Medicine’s *Crossing the Quality Chasm* report, which states that care should not just occur with face-to-face visits, but that continuous “access to care should be provided over the Internet” [6] and that meaningful use of health information technology should be implemented [7]. However, some patients may choose not to engage in Web-based health care and others may be unable. Older patients, ethnic and racial minorities, and those with lower education levels or who are unemployed have less access to the Web, typically described as the “digital divide” [8-11]. Other patients with Web access might be healthier than those without access, potentially increasing health outcome disparities.

The Electronic Communications and Home Blood Pressure Monitoring (e-BP) study was a randomized controlled trial designed to test whether use of home blood pressure (BP) monitoring, use of an existing patient Web portal with a patient-shared electronic health record and secure email, and Web-based pharmacist-assisted care led to hypertension control. During recruitment we attempted to contact all patients with hypertension from 10 clinics to determine whether they were eligible and willing to participate. Patients randomized to home BP monitoring and Web-based collaborative care with a pharmacist were almost twice as likely as those in usual care to have controlled BP at the 12-month follow-up visit (adjusted relative risk [RR] 1.84, 95% CI 1.48-2.29) [2]. We describe here characteristics of patients with hypertension who were not eligible to participate because of lack of computer access. Of those with computer access, we also compared characteristics of patients with controlled and uncontrolled hypertension. Identifying characteristics of these populations will provide a context for engaging participation in and designing future Web-based interventions that lead to improved health outcomes for all populations. 

## Methods

We attempted to contact all patients with a diagnosis of hypertension and taking medications for this from 10 primary care clinics to invite them to participate in the e-BP trial. During recruitment patients could refuse to participate (either actively or passively, by not responding) or be ineligible to participate because of lack of computer access, having controlled BP, or having other ineligibility medical conditions. We attempted to survey all patients contacted regardless of their willingness and eligibility to participate in the e-BP trial. Eligible patients who provided consent were randomly assigned to (1) usual care, (2) receive a home BP monitor and training to use it, and training to use an existing patient Web portal with secure messaging and other Web services, or (3) group 2 interventions plus collaborative pharmacist care management delivered via Web communications. The study design was based on the chronic care model [12]. A complete description of the methods and recruitment processes of the e-BP study were reported elsewhere, but an overview follows [13].

### Study Setting

We recruited participants between June 2005 and December 2006 at 10 primary care medical centers within Group Health, a nonprofit, integrated group practice that provides both medical coverage and care to more than 600,000 residents of Washington State and Idaho. Group Health Research Institute’s Institutional Review Board reviewed and approved this study.

Group Health has a comprehensive electronic health record system, EpicCare (Epic Systems Corporation, Verona, Wisconsin, USA), which integrates clinical communication and information processes into a single interface that includes physician order entry (eg, laboratory tests, prescriptions, and referrals), documentation of all patient encounters, clinical decision support, clinical messaging between physicians, secure online messaging with patients, and automated reminders at the point of care. Group Health provides patients with access to the electronic health record via a patient Web site (myGroupHealth), which they can use to refill medications, make appointments, view portions of their medical record (current health conditions, laboratory test results, after-visit summaries, allergies, and medications), and send secure messages to their health care team. Detailed description of the patient Website and its integration into overall access to care at Group Health is described elsewhere [14,15].

### Recruitment

We used clinical and administrative data routinely collected and maintained by Group Health to identify all patients age 25-75 years with a diagnosis of hypertension and taking antihypertensive medications, with no diagnoses of diabetes, cardiovascular or renal disease, or other serious conditions (such as dementia or active treatment of cancer). Research assistants telephoned potential participants to confirm eligibility, including computer access (defined as access to a computer, the Internet, and an email address), and willingness to attend screening visits. All patients surveyed by telephone, including those ineligible or refusing to participate in the study, were asked to answer several demographic questions (race and ethnicity, education level, occupation), computer access questions, and whether they owned a home BP monitor.  

Patients with a hypertension diagnosis, computer access, and no other exclusions were invited to an in-person screening visit at their primary care medical center to obtain BP measurements. Patients who had not previously signed up to use the myGroupHealth patient Website secure services were assisted in doing so and given Group Health pamphlets on the various functionalities of the Web portal. Patients were eligible to participate in the trial if their BP was elevated at both of two in-person screening visits. BP was measured three times at each visit using a validated Omron Hem-705CP automated monitor (OMRON Corporation, Schaumburg, IL, USA) with a cuff fitted for the patient’s upper arm circumference [16]. The first measurement was dropped and the last two were averaged. If the mean diastolic BP was 90-109 mmHg or systolic BP was 140-199 mmHg at both visits, the patient was invited to participate, and written informed consent was obtained. Patients were randomly assigned to one of three study conditions. Group 1 (usual care) received Group Health’s pamphlet on elevated BP and were advised to work with their doctor to improve their BP control. Group 2 (Web only) received a home BP monitor and training to use it proficiently on their own and a tour of the functionalities of the myGroupHealth Website. Group 3 (Web plus pharmacist) was the same as group 2 plus Web-based pharmacist collaborative care. Intervention components are described in more detail elsewhere [2,13].

### Measures

We used automated databases to obtain sex, age, insurance plan type (commercial, Medicare, Medicaid, or state-subsidized basic health), prior use of secure messaging, and body mass index (BMI) using the most recently recorded weight and height. The Johns Hopkins Adjusted Clinical Group’s case-mix system was used to measure each individual’s overall level of morbidity burden. Their software assigns each individual a level of overall morbidity depending on age, sex, and number and types *International Classification of Diseases*, ninth revision, codes over a 12-month period [17,18]. Patients were classified as having high, medium, or low expected clinical need. Demographic variables not available in the Group Health databases, including education level, employment status, marital status, and race, were collected during the telephone survey. When participants chose more than one category for race, coding precedence was given to Hispanic, non-Hispanic black, Asian, other, and non-Hispanic white categories, in that order. Survey participants were also asked if they used a home BP monitor.

### Statistical Analysis

We present frequencies of patient characteristics by four recruitment outcomes (unable to contact, refused, ineligible, and randomized) and applied Pearson chi-square tests to assess any differences between groups. To evaluate factors related to computer access we used generalized linear models with a log link and robust sandwich variance estimator using a modified Poisson regression framework to estimate RR of not having computer access [19]. Logistic regression models were not used because computer access was not rare. We present two sets of adjusted RRs: (1) adjusted for age and sex only, and (2) adjusted for all variables shown in Table 2 including age, sex, socioeconomic measures, BMI, expected clinical need, and having a home BP monitor. 

Modified Poisson regression models were also used to estimate the RR of uncontrolled BP among participants attending the screening visits. We present two sets of adjusted RRs for uncontrolled BP: (1) adjusted for age and sex only, and (2) adjusted for age, sex, education, race, and BMI. In our full model, we adjusted only for covariates that were associated with the uncontrolled BP in the first model. The primary analysis defined BP control based on the BP measurement from the first screening visit. A sensitivity analysis was also performed using a more conservative definition of uncontrolled BP based on study recruitment guidelines requiring uncontrolled BP at both screening visits.

Medicare insurance was omitted from multivariable models including both insurance type and age because of the significant overlap with the age category 65-75 years. In models estimating the RR of uncontrolled BP, the employment categories disabled, unemployed, and other were combined due to small sample sizes.

All analyses were performed using Stata version 11.0 statistical software (StataCorp LP, College Station, TX, USA). All reported *P*-values and 95% CIs are two sided with significance defined at the 0.05 alpha level and are based on the Wald statistic unless otherwise specified.

## Results

**Figure 1:**
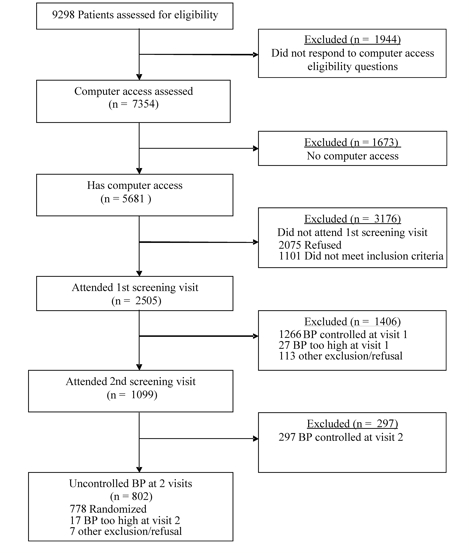
Recruitment Flow

Our recruitment sample (N = 9298) included all patients age 25-74 years from 10 primary care medical centers with administrative data indicating they had a diagnosis of hypertension, were taking antihypertensive medications, and had no exclusionary conditions (Figure 1). Automated data were available on all 9298 hypertension patients who were sent invitation letters. Of the 8840 (95.1%) patients we were able to contact, 83.2% (7354/8840) responded to the survey questions assessing computer access eligibility, including 71.0% (2153/3032) of those who refused participation in the trial.

### Refusal and Ineligibility to Participate

Of those we attempted to contact, 32.6% (3032/9298) refused participation, 2598 at the time of the telephone survey and 434 after agreeing to attend a screening visit (either by not attending or by refusing at the time of the screening visit) (Table 1). Compared to trial participants, patients refusing participation were significantly more likely to be female (*P* = .002), to be younger (*P* = .002), to be from a racial or ethnic minority group (*P* < .001), and to have lower levels of education (*P* = .002). The most common reasons for refusal were either being too busy or not being interested in participating. Only 15% (447/3032) listed unwillingness to use the patient Web portal as a reason for refusal. 

Over half of the sampled patients (5030/9298, 54%) were ineligible for the study. The most common reasons for ineligibility were lack of computer access (n = 1673), and controlled BP at either the first or second screening visit (n = 1563). If patients lacked computer access they were not invited to have screening BP visits. Thus, patients might have had more than one reason for exclusion that was not ascertained. The demographic characteristics of ineligible patients differed by reason for ineligibility; therefore, we separately examined characteristics associated with the two most common reasons for eligibility, lack of computer access and controlled BP. 

**Table 1:** Demographic characteristics by recruitment outcome (N = 9298)

		Unable to contact		Refused		Ineligible		Randomized	
		n = 458		n = 3032		n = 5030		n = 778	
		n	%	n	%	n	%	n	%
Sex (% female)		246	53.7	1638	54.0^a^	3040	60.4^a^	372	47.8
**Age (years)**									
	25-39	22	4.8^a^	109	3.6^a^	137	2.7^a^	13	1.7
	40-54	191	41.7	906	29.9	1322	26.3	214	27.5
	55-64	169	36.9	1319	43.5	1951	38.8	334	42.9
	65-75	76	16.6	698	23.0	1620	32.2	217	27.9
**BMI^b^ (kg/m^2^)**									
	Normal/low (<25)	58	17.0^a^	430	16.7^a^	728	17.6^a^	67	9.5
	Overweight (25-30)	122	35.8	885	34.3	1375	33.3	227	32.1
	Obese (≥30)	161	47.2	1268	49.1	2026	49.1	414	58.5
	Missing^c^	117	(25.6)	449	(14.8)	901	(17.9)	70	(9.0)
**Insurance product**									
	Commercial	379	82.8^a^	2328	76.8^a^	3362	66.8^a^	574	73.8
	Medicare	66	14.4	661	21.8	1529	30.4	200	25.7
	Basic health/Medicaid	13	2.8	43	1.4	139	2.8	4	0.5
**Expected clinical need**									
	Low	156	35.1^a^	632	21.1	809	17.4^a^	145	18.7
	Medium	232	52.3	1819	60.7	2803	60.4	507	65.3
	High	56	12.6	544	18.2	1032	22.2	124	16.0
	Missing^c^	14	(3.1)	37	(1.2)	386	(7.7)	2	(0.3)
**Prior use of secure messaging**									
	(% yes)	101	22.1^a^	1063	35.1^a^	1430	28.4^a^	338	43.4
**Race**		NA^d^						
	White, non-Hispanic			1592	74.6^a^	3335	77.3^a^	637	82.0
	Black, non-Hispanic			178	8.3	297	6.9	60	7.7
	Hispanic			59	2.8	122	2.8	16	2.1
	Asian			159	7.5	294	6.8	28	3.6
	Other			147	6.9	264	6.1	36	4.6
	Missing^c^			897	(29.6)	718	(14.3)	1	(0.1)
**Education**		NA^d^						
	<HS^e^ graduate			24	1.1^a^	129	3.0^a^	5	0.6
	HS graduate/GED^f^			257	12.1	672	15.6	57	7.3
	Some post-HS			855	40.1	1723	39.9	324	41.7
	College graduate			511	24.0	961	22.3	195	25.1
	Postgraduate			485	22.8	834	19.3	197	25.3
	Missing^c^			900	(29.7)	711	(14.1)	0	(0.0)

**Employment**		NA^d^						
	Full-time			1268	59.4	2050	47.4 ^a^	435	56.0
	Retired			624	29.2	1686	39.0	270	34.8
	Part-time			153	7.2	324	7.5	51	6.6
	Disabled			23	1.1	68	1.6	4	0.5
	Unemployed			22	1.0	63	1.5	7	0.9
	Other			44	2.1	133	3.1	10	1.3
	Missing^c^			898	(29.6)	706	(14.0)	1	(0.1)
**Home blood pressure monitor**		N/A^d^						
	Yes			1452	67.6^a^	2533	58.4	437	56.2
	No			697	32.4	1808	41.7	341	43.8
	Missing^c^			883	(29.1)	689	(13.7)	0	(0.0)

^a^ *P* < .05, compared to randomized group.

^b^ BMI: body mass index.

^c^ Percentages with missing data (in parentheses) are not included in column percentages.

^d^ NA: not available – survey data not collected from patients we were unable to contact.

^e^ HS: high school.

^f^ GED: general equivalency diploma.

### Computer Access

The majority (7354/8840, 83.2%) of patients we contacted were willing to answer questions on computer access, even those who refused to participate in the study (2153/3032, 71%). Of those answering the computer questions, 22.8% (1673/7354) lacked computer access (no access to a computer, the Web, or email) (Table 2). The RR for lack of computer access was 2.63 (CI 2.30-3.01) for those with a high school diploma and 3.62 (CI 3.05-4.29) for those with less than a high school diploma compared to college graduates. There was a similar relationship between age and lack of computer access. Those ages 65-75 years were two times more likely to not have computer access compared to those ages 40-54 (RR 2.37, CI 2.11-2.67). Being any race or ethnicity other than white was also associated with increased risk for not having computer access, as was being disabled or unemployed, and having Medicaid or state-supported insurance. Age, race-ethnicity, employment, and insurance associations were not attenuated by controlling for education or other covariates. Patients without home BP monitors (at baseline) were also more likely to not have computer access (RR 1.32, CI 1.21-1.44). Anticipated clinical need was not associated with computer access.

Having computer access did not guarantee participation. Almost 40% (2152/5681, 37.9%) of patients with computer access refused participation. Similar to those who refused overall, computer-able refusers were significantly more likely to be female (*P* < .001), younger (*P* = .008), nonwhite (*P* < .001), and less educated (*P* = .002) than those randomized to participate in the study. They also were less likely to have used secure messaging (*P* = .01) and own a home BP monitor (*P* < .001). Even though the majority of people with computer access agreed to go on with the recruitment process, 78.0% (2751/3529) were not eligible, mainly because of controlled BP, discussed in more detail below. 

**Table 2:** Adjusted relative risk (RR) of not having computer access by demographic characteristics among patients for whom computer access was ascertained during the telephone screening survey (n = 7354)

		Access		No Access		Adjusted for age and sex		Adjusted for all variables^a^	
		n	Row %	n	Row %	RR	95% CI	RR	95% CI
Total		5681	77.3	1673	22.8				
**Sex**									
	Female	3207	75.5	1042	24.5	1.00	Referent	1.00	Referent
	Male	2474	79.7	631	20.3	0.85	0.78-0.92	1.01	0.91-1.11
**Age (years)**									
	25-39	168	87.1	25	13.0	0.87	0.59-1.26	0.89	0.58-1.36
	40-54	1675	84.8	300	15.2	1.00	Referent	1.00	Referent
	55-64	2437	81.4	557	18.6	1.23	1.08-1.40	1.33	1.15-1.54
	65-75	1401	63.9	791	36.1	2.37	2.11-2.67	2.27	1.92-2.67
**BMI^b^ (kg/m^2^)**									
	Normal/low (<25)	750	73.8	266	26.2	1.00	Referent	1.00	Referent
	Overweight (25-30)	1646	77.5	478	22.5	0.93	0.81-1.05	0.92	0.81-1.04
	Obese (≥30)	2543	78.7	687	21.3	0.96	0.85-1.08	0.91	0.80-1.03
**Insurance product**									
	Commercial	4292	83.1	872	16.9	1.00	Referent	1.00	Referent
	Basic health/Medicaid	58	52.7	52	47.3	2.74	2.24-3.35	1.98	1.52-2.59
**Expected clinical need**									
	Low	1006	77.1	299	22.9	1.00	Referent	1.00	Referent
	Medium	3456	78.9	924	21.1	0.83	0.74-0.93	0.83	0.73-0.95
	High	1073	73.2	394	26.8	0.96	0.85-1.09	0.94	0.82-1.09
**Race**									
	White, non-Hispanic	4483	80.5	1085	19.5	1.00	Referent	1.00	Referent
	Black, non-Hispanic	398	74.4	137	25.6	1.56	1.34-1.81	1.38	1.17-1.62
	Hispanic	138	70.1	59	30.0	1.81	1.47-2.23	1.58	1.26-1.99
	Asian	320	66.5	161	33.5	1.86	1.63-2.12	1.96	1.70-2.27
	Other	315	70.2	134	29.8	1.60	1.38-1.85	1.42	1.22-1.66
**Education**									
	<HS^c^ graduate	57	35.9	102	64.2	3.62	3.05-4.29	3.22	2.67-3.87
	HS graduate/GED^d^	560	56.7	427	43.3	2.63	2.30-3.01	2.53	2.18-2.93
	Some post-HS	2222	76.5	681	23.5	1.55	1.36-1.77	1.56	1.36-1.80
	College graduate	1421	85.2	247	14.8	1.00	Referent	1.00	Referent
	Postgraduate	1401	92.9	117	7.7	0.51	0.41-0.62	0.53	0.42-0.66
**Employment**									
	Full-time	3184	84.8	571	15.2	1.00	Referent	1.00	Referent
	Retired	1806	70.0	776	30.1	1.26	1.11-1.42	1.18	1.04-1.34
	Part-time	423	80.0	106	20.0	1.14	0.95-1.37	1.12	0.92-1.37
	Disabled	56	59.0	39	41.1	2.60	2.03-3.33	1.84	1.41-2.40
	Unemployed	65	69.9	28	30.1	1.95	1.44-2.66	1.41	1.00-1.99
	Other	129	69.0	58	31.0	1.72	1.38-2.16	1.22	0.95-1.56
**Home blood pressure monitor**									
	Yes	3527	79.7	897	20.3	1.00	Referent	1.00	Referent
	No	2150	75.4	700	24.6	1.32	1.21-1.44	1.26	1.15-1.38

^a^ All variables shown in this table are included in the model.

^b^ BMI: body mass index.

^c^ HS: high school.

^d^ GED: general equivalency diploma.

### Blood Pressure Control

After the telephone survey, 2937 hypertensive patients with computer access agreed to attend a screening visit to have their BP measured to verify eligibility (uncontrolled BP). Of these, 2505 patients attended the first screening visit (Table 3), where 49.5% (1239/2505) had uncontrolled BP and were invited to a second screening visit. Using our stricter definition of uncontrolled BP at two screening visits, only 33.9% (802/2365) of the patients who completed screening had uncontrolled BP (Table 4). We were unable to determine BP control status for 134 patients who had uncontrolled BP at the first screening visit but did not attend the second visit. This group was excluded from the sensitivity analysis, which used the more strict definition requiring two measures to verify uncontrolled BP.

Male sex, non-Hispanic black race, and being overweight or obese were risk factors for uncontrolled BP regardless of whether uncontrolled BP was defined based on a single screening visit (Table 3) or on two screening visits (Table 4). These risks were somewhat more pronounced when we used the stricter study definition for uncontrolled BP. In the primary analysis, adjusted RR for uncontrolled BP for obese patients was 1.60 (CI 1.28-2.00) when compared to normal-weight individuals in the model that included age, sex, education, race, and BMI. Patients who reported not having a home BP monitor had a marginally higher risk of uncontrolled BP, with the RR attenuating in the fully adjusted models. Expected clinical need was not related to BP control.

Among patients attending at least one screening visit, 44 had severe hypertension with BP too high to be eligible to participate in the trial (defined as an average systolic BP ≥ 200 mmHg or diastolic BP ≥ 110 mmHg; data not shown). Compared to those enrolled with uncontrolled BP, ineligible patients with very high BPs were significantly more likely to be less than age 55 years (61.4% [27/44] vs 34.6% [269/778], *P* < .001) and non-Hispanic black (15.9% [7/44] vs 7.7% [60/778], *P* = .05). However, they did not differ in level of obesity (*P* = .56).

**Table 3:** Adjusted relative risk (RR) of uncontrolled blood pressure (BP) among patients completing the first screening visit (n = 2505)

		Controlled BP^a^		Uncontrolled BP^a^		Adjusted for age and sex		Adjusted for age, sex, education, race, and BMI^b^	
		n	Row %	N	Row %	RR	95% CI	RR	95% CI
Total		1266	50.4	1239	49.5				
Systolic BP^a^ (mmHg), mean (SD)		126.3 (8.4)		151.1 (12.4)			
Diastolic BP^a^ (mmHg), mean (SD)		77.7 (7.2)		89.3 (9.2)			
**Sex**									
	Female	795	55.8	630	44.2	1.00	Referent	1.00	Referent
	Male	471	43.6	609	56.4	1.28	1.18-1.38	1.29	1.19-1.40
**Age (years)**									
	25-39	24	51.1	23	48.9	1.00	0.73-1.36	0.90	0.65-1.26
	40-54	374	52.3	341	47.7	1.00	Referent	1.00	Referent
	55-64	544	50.3	537	49.7	1.04	0.94-1.14	1.04	0.94-1.15
	65-75	324	48.9	338	51.1	1.08	0.97-1.20	1.14	1.02-1.27
**BMI (kg/m^2^)**									
	Normal (<25)	215	65.0	116	35.1	1.00	Referent	1.00	Referent
	Overweight (25-30)	391	50.9	377	49.1	1.36	1.16-1.61	1.34	1.14-1.58
	Obese (≥30)	537	46.1	628	53.9	1.53	1.31-1.79	1.47	1.25-1.72
**Insurance product**									
	Commercial	950	50.8	919	49.2	1.00	Referent	1.00	Referent
	Basic health/Medicaid	7	43.8	9	56.3	1.10	0.71-1.69	1.02	0.60-1.75
**Expected clinical need**									
	Low	229	49.5	234	50.5	1.00	Referent	1.00	Referent
	Medium	814	50.5	797	49.5	0.99	0.90-1.10	0.99	0.89-1.11
	High	218	51.5	205	48.5	0.98	0.86-1.12	0.96	0.84-1.11
**Prior use of secure messaging**									
	No	664	48.4	708	51.6	1.00	Referent	1.00	Referent
	Yes	602	53.1	531	46.9	0.92	0.85, 0.99	0.99	0.91, 1.07
**Race**									
	White, non-Hispanic	1067	51.4	1011	48.7	1.00	Referent	1.00	Referent
	Black, non-Hispanic	65	42.2	89	57.8	1.22	1.06-1.40	1.26	1.10-1.45
	Hispanic	25	47.2	28	52.8	1.09	0.84-1.41	1.12	0.86-1.47
	Asian	63	59.4	43	40.6	0.85	0.67-1.08	0.95	0.74-1.22
	Other	43	39.8	65	60.2	1.22	1.04-1.43	1.18	1.00-1.29
**Education**									
	<HS^c^ graduate	10	52.6	9	47.4	0.98	0.59-1.64	0.94	0.53-1.64
	HS graduate/GED^d^	97	47.8	106	52.2	1.13	0.97-1.32	1.10	0.94-1.29
	Some post-HS	436	46.8	496	53.2	1.15	1.04-1.27	1.10	0.99-1.22
	College graduate	339	52.4	308	47.6	1.00	Referent	1.00	Referent
	Postgraduate	384	54.6	320	45.5	0.94	0.84-1.06	0.95	0.84-1.07

**Employment**									
	Full-time	690	49.9	692	50.1	1.00	Referent	1.00	Referent
	Retired	429	49.9	430	50.1	0.98	0.88-1.09	0.98	0.88-1.10
	Part-time	101	56.4	78	43.5	0.91	0.77-1.09	0.94	0.78-1.14
	Other	46	54.8	38	45.2	0.97	0.76-1.23	0.89	0.68-1.17
**Home BP monitor**									
	Yes	747	51.6	702	48.5	1.00	Referent	1.00	Referent
	No	518	49.2	536	50.9	1.07	0.99-1.16	1.03	0.94-1.12

^a^ BP and BP control measured at the first screening visit.

^b^ BMI: body mass index.

^c^ HS: high school.

^d^ GED: general equivalency diploma.

**Table 4:** Adjusted relative risk (RR) of uncontrolled blood pressure (BP) based on study recruitment guidelines requiring two measures to define uncontrolled BP (n = 2365)

		Controlled BP^a^		Uncontrolled BP^a^		Adjusted for age and sex		Adjusted for age, sex, education, race, and BMI^b^	
		n	Row %	n	Row %	RR	95% CI	RR	95% CI
Total		1563	66.1	802	33.9				
Systolic BP^c^ (mmHg), mean (SD)		129.9 (11.2)		152.8 (11.7)			
Diastolic BP^c^ (mmHg), mean (SD)		79.4 (8.0)		89.7 (8.7)			
**Sex**									
	Female	966	71.5	386	28.6	1.00	Referent	1.00	Referent
	Male	597	58.9	416	41.1	1.44	1.29-1.62	1.50	1.33-1.69
**Age (years)**									
	25-39	27	67.5	13	32.5	0.93	0.59-1.48	0.78	0.48-1.29
	40-54	450	66.9	223	33.2	1.00	Referent	1.00	Referent
	55-64	678	66.3	345	33.7	1.01	0.88-1.16	1.04	0.90-1.20
	65-75	408	64.9	221	35.1	1.08	0.93-1.25	1.15	0.99-1.35
**BMI (kg/m^2^)**									
	Underweight/normal (<25)	244	77.5	71	22.5	1.00	Referent	1.00	Referent
	Overweight (25-30)	499	68.2	233	31.8	1.34	1.07-1.69	1.31	1.04-1.64
	Obese (≥30)	670	61.1	427	38.9	1.67	1.35-2.08	1.60	1.28-2.00
**Insurance product**									
	Commercial	1169	66.3	594	33.7	1.00	Referent	1.00	Referent
	Basic health/Medicaid	394	65.5	208	34.6	0.86	0.38-1.92	0.62	0.18-2.20
**Expected clinical need**									
	Low	290	66.2	148	33.8	1.00	Referent	1.00	Referent
	Medium	1003	65.8	522	34.2	1.04	0.89-1.20	0.96	0.82-1.12
	High	265	67.1	130	32.9	1.01	0.84-1.23	0.95	0.78-1.16
**Prior use of secure messaging**									
	No	831	64.5	458	35.5	1.00	Referent	1.00	Referent
	Yes	732	68.0	344	32.0	0.91	0.81-1.02	1.00	0.88-1.12
**Race**									
	White, non-Hispanic	1321	66.9	654	33.1	1.00	Referent	1.00	Referent
	Black, non-Hispanic	76	54.3	64	45.7	1.43	1.18-1.74	1.52	1.26-1.83
	Hispanic	33	66.0	17	34.0	1.03	0.69-1.52	1.01	0.66-1.55
	Asian	72	71.3	29	28.7	0.89	0.65-1.22	1.05	0.76-1.45
	Other	57	60.6	37	39.4	1.17	0.90-1.52	1.15	0.88-1.49
**Education**									
	<HS^d^ graduate	13	72.2	5	27.8	0.83	0.38-1.84	0.87	0.40-1.88
	HS graduate/GED^e^	125	67.2	61	32.8	1.08	0.85-1.36	1.06	0.83-1.35
	Some post-HS	544	61.8	336	38.2	1.23	1.07-1.42	1.17	1.01-1.35
	College graduate	416	67.8	198	32.3	1.00	Referent	1.00	Referent
	Postgraduate	465	69.7	202	30.3	0.92	0.79-1.09	0.95	0.80-1.12
**Employment**									
	Full-time	850	65.3	452	34.7	1.00	Referent	1.00	Referent
	Retired	539	66.3	274	33.7	0.95	0.81-1.11	0.99	0.84-1.16
	Part-time	117	68.4	54	31.6	0.98	0.78-1.24	1.06	0.83-1.35
	Other	57	73.1	21	26.9	0.86	0.59-1.24	0.80	0.53-1.21
**Home BP Monitor**									
	Yes	928	67.4	449	32.6	1.00	Referent	1.00	Referent
	No	633	64.2	353	35.8	1.12	1.00-1.26	1.06	0.94-1.19

^a^ BP control based on study recruitment guidelines requiring two measures (visits) to define controlled and uncontrolled BP.

^b^ BMI: body mass index.

^c^ BP measured at the first screening visit.

^d^ HS: high school.

^e^ GED: general equivalency diploma.

## Discussion

Patient-shared electronic health records and secure Web communications allow new opportunities for patients to be uniquely involved in their own care, including viewing their medical records, communicating asynchronously by secure email, and receiving other Web-based services. The e-BP trial demonstrated that the use of these tools and Web-based collaborative pharmacist care led to significant decreases in both systolic and diastolic BP and improved BP control.

Our recruitment efforts included contacting almost all patients with a hypertension diagnosis from 10 primary care clinics. The majority of people we contacted were interested in continuing with the recruitment process; however, one third declined. Those refusing were more likely to be from racial minority and lower socioeconomic groups. Difficulty recruiting from underserved and minority groups has been documented [20,21]. Enrolling people in Web-related research poses additional challenges, as the same groups that have been less likely to participate in clinical trials are also less likely to have computer access. 

In 2005 and 2006, over 20% of the patients we attempted to recruit could not participate in a Web-based intervention because of lack of computer access. Lack of computer access was strongly related to lower levels of education, older age, and minority race and ethnicity. Adjustments for potential confounders made little difference. These groups are those typically described as being part of the “digital divide.” Multiple observational studies have documented age, race, socioeconomic, and educational disparities in the use of patient electronic health records and eHealth services [22-24]. These same groups are more likely to experience disparities in health access and outcomes. Blacks, on average, die 6 years earlier than whites from heart disease [25]. Paradoxically, those who might benefit the most from eHealth innovations may be less able or unwilling to use these resources. Eysenbach has called this association between vulnerable populations and lack of computer and information access *“the inverse information law”*: Access to health information is often most difficult for those who need it most [10].

Interestingly in our analysis, expected clinical need was not related to refusal, lack of computer access, or BP control. Others have found no or increased associations between comorbidity and health status, and Internet and use of patient electronic health records. Ralston et al [26] and Weppner et al [27] found increased use of secure messaging in those with the highest levels of comorbidity. Gracia and Herrero [28] found that, once socioeconomic factors were controlled for, older adults (age 55-74 years) with poor self-reported health were more likely to use the Internet. 

Over half of the patients we attempted to recruit had controlled BP and did not need a pharmacist’s intervention. Using the stricter criteria of uncontrolled BP at two separate visits, 66.1% (1563/2365) of the patients had controlled BP, compared to 52.1% (1304/2505) at a single visit. After the diagnosis of hypertension is established, medication decisions are often based on measurements at a single office visit, which according to our findings might lead to misclassifying many people as having uncontrolled BP. While there is a direct relationship between increasing systolic BP and cardiovascular disease events [29], there is no evidence for those with essential hypertension that lowering BP ≤ 140/90 mmHg leads to improved outcomes. Misclassifying people as having uncontrolled BP could result in harm and unnecessary costs [30]. At the time the study was conducted, the patient-shared electronic health record had just been implemented, and there were not enough BP data to prescreen participants. Over 98% of Group Health patients with a primary care visit have at least one BP measure in their electronic health record in any given year. Automated data now could be used to more efficiently identify patients with uncontrolled BP.

Concordant with the literature, non-Hispanic blacks were more likely than other racial and ethnic groups to have uncontrolled BP [31]. Obese patients were also more likely to have uncontrolled BP. Only 7.2% of the trial participants with uncontrolled BP had a normal BMI (using baseline clinical measurements). Obesity is a known risk factor for hypertension incidence and uncontrolled BP [32]. Obesity is also more common in those from minority racial and ethnic groups, and with lower income and lower levels of education [33]. Despite these relationships, in our analysis obesity was not related to either refusing to participate or lack of computer access. Patients were not assessed for metabolic syndrome and sleep apnea, likely contributory factors to uncontrolled BP. Our finding that men were more likely to have uncontrolled BP has also been cited in the literature [34]. The mechanisms for these differences are not well understood. Others have reported higher incidence of uncontrolled BP in women, but generally in older populations [35,36]. Level of education and expected clinical need were not related, and age was only weakly related, to BP control. The association between these covariates and BP control might have changed had we invited those without computer access to attend screening visits. 

Our analysis has several important limitations. Almost 21% of the patients we attempted to contact did not answer the survey questions, and we have no information on race, education level, self-monitoring, computer access, or BP control for this group. Additionally, almost all patients at Group Health have health insurance, few have Medicaid, and our results may not be representative of populations without health insurance. Additionally, the Pacific Northwest is known for being “wired” and potential eHealth-associated disparities may be greater in other communities [37]. 

A particular strength of our analysis is that we were able to collect administrative and electronic medical record data on the entire recruitment sample. Of those successfully contacted (8840/9298, 95.1%), over 80% (7354/8840, 83.2%) consented to answering a brief nonparticipant questionnaire. Few trials, including hypertension and eHealth studies, have access to nonparticipant data. In the Antihypertensive and Lipid-lowering Treatment to Prevent Heart Attack Trial (ALLHAT) over one third of the 33,357 participants in the hypertension trial component were black; however, because recruitment occurred by a variety of methods (radio and newspaper ads, letters, flyers, referral), the researchers were unable to characterize eligible nonparticipants. Glasgow et al [38], in a Web-based weight-loss intervention, found that people age 60 years and older were less likely to enroll, but did not have data for race or education. Stopponi et al [39], in a Web-based nutrition trial, imputed education and income level by census tract. Similar to our results, their results showed that nonparticipants were more likely to be less educated and older. Our analysis adds to these studies, by systematically attempting to invite all patients with hypertension to participate and by capturing a richer set of data. Additional information on type of Internet connection, proficiency with, time spent on, and different usages of the Internet, and their perceptions of Web-based care would have provided further insight, but we were limited in the number of questions we were allowed to ask patients who refused further participation in the recruitment process. 

Over 65% of adults who receive care at Group Health clinics are registered and have access to their patient-shared electronic health record and comprehensive Web services, and 30.7% of outpatient primary care encounters occur virtually, over the Web (with phone visits at 15.3% and in-person visits, 54.0%, accounting for the rest) [40]. Patients are very satisfied with these services, particularly secure email, medical test results, and medication refill services [14]. In contrast, only a small proportion of the US population has access to an electronic health record; however, in surveys, most would like access [41,42]. 

Patient Web portals will likely be increasingly available in other media forms, such as cell phones. In 2008, 84% of American adults owned a cell phone, compared to 74% having access to the Internet [43]. Web communications also have the potential to be translated into different languages, adapted to different literacy levels, and used by people with physical disabilities, which over time might help to mitigate disparity gaps. Patient Web portals also may lead to decreased health care utilization and costs. After the introduction of a patient Web portal in Kaiser Permanente, there was a 20% decrease in primary care and specialty care visits [5,44]. For these reasons and the success of the e-BP trial, we believe that increasing the availability of Web portals is warranted. However, our data show that it is necessary to ensure equity for those without access. 

Systematic reviews and meta-analyses have found strong evidence that “team-based” care for hypertension (care provided by a health professional such as a pharmacist or nurse separate from office visits) improves BP control [45,46]. Successful studies have been conducted in a variety of settings (clinic, worksite, and community facilities) and have used different communication techniques (face-to-face visits, telephone, or facilitated transfer of data), and use of e-communication is only one of many different effective options. Which type of program offered could be based on the targeted population, local resources, satisfaction, and costs.

In conclusion, patients unwilling or unable to participate because of lack of computer access in a Web-based intervention to improve hypertension control were more likely to be from populations that already experience disparities in health care. The majority of those willing and able to receive Web-based care had controlled BP and did not need additional Web-based pharmacist medication management. As we strive to learn how best to use patient-shared electronic health records with Web communications to improve the care of chronic conditions, specific attention will be required to insure that health disparities are minimized.

## Abbreviations

ALLHAT: Antihypertensive and Lipid-lowering Treatment to Prevent Heart Attack Trial
BMI: body mass index
BP: blood pressure
e-BP: Electronic Communications and Home Blood Pressure Monitoring
RR: relative risk
